# Most prominent challenges in translational neuroscience and strategic solutions to bridge the gaps: Perspectives from an editorial board interrogation

**DOI:** 10.37349/en.2025.1006106

**Published:** 2025-08-12

**Authors:** Dirk M. Hermann, Marco Bacigaluppi, Claudio L. Bassetti, Gabrio Bassotti, Johannes Boltze, Andrew Chan, Turgay Dalkara, Adam Denes, Exuperio Diez-Tejedor, Richard Dodel, Thorsten R. Doeppner, Egor Dzyubenko, Ayman ElAli, Tamas Fulop, Alexander Gerhard, Bernd Giebel, Janine Gronewold, Matthias Gunzer, Thomas Heinbockel, Kaibin Huang, Marcello Iriti, Hans-Otto Karnath, Kasteleijn-Nolst Trenite, Ertugrul Kilic, Giuseppe Lanza, Arthur Liesz, Tim Ullrich Magnus, Jessica Mandrioli, Ayan Mohamud-Yusuf, Thomas Müller, Suyue Pan, Luca Peruzzotti-Jametti, Stefano Pluchino, Ryszard Pluta, Aurel Popa-Wagner, Ameneh Rezayof, Mohamed L. Seghier, Xinhua Shu, Vikram Singh, Jussi Sipilä, Mark Slevin, Yamei Tang, Georgios Tsivgoulis, Giustino Varrassi, Chen Wang, Bayram Yilmaz, Maha S. Zaki, Jinwei Zhang

**Affiliations:** 1 Chair of Vascular Neurology, Dementia and Aging, Department of Neurology, University Hospital Essen, University of Duisburg-Essen, 45147 Essen, Germany; 2 Center for Translational Neuro- and Behavioral Sciences (C-TNBS), University Hospital Essen, University of Duisburg-Essen, 45147 Essen, Germany; 3 Center for Molecular Cardiology, University of Zurich, 8952 Zurich, Switzerland; 4 Department of Neurology, San Raffaele Hospital, 20132 Milan, Italy; 5 Department of Neurology, Medical Faculty, Inselgruppe, Bern University Hospital, University of Bern, 3010 Bern, Switzerland; 6 Department of Medicine and Surgery, Perugia University School of Medicine, 06132 Perugia, Italy; 7 School of Life Sciences, University of Warwick, CV4 7AL Coventry, UK; 8 Department of Neuroscience, Bilkent University, 06800 Ankara, Turkey; 9 Laboratory of Neuroimmunology, HUN-REN Institute of Experimental Medicine, H-1083 Budapest, Hungary; 10 Department of Neurology, IdiPAZ, La Paz University Hospital, Universidad Autonoma de Madrid, 28046 Madrid, Spain; 11 Department of Geriatrics, University of Duisburg-Essen, 45141 Essen, Germany; 12 Department of Neurology, University Hospital Gießen and Marburg, Justus Liebig University Gießen, 35392 Gießen, Germany; 13 Department of Psychiatry and Neuroscience, Université Laval, Quebec City, QC G1V 4G2, Canada; 14 Departments of Medicine and Radiology and Nuclear Medicine, Université de Sherbrooke, Sherbrooke, QC J1H 5H3, Canada; 15 Division of Psychology, Communication and Human Neuroscience, Wolfson Molecular Imaging Centre, University of Manchester, M13 9PL Manchester, UK; 16 Department of Nuclear Medicine, University Hospital Essen, University of Duisburg-Essen, 45147 Essen, Germany; 17 Institute for Transfusion Medicine, University Hospital Essen, University of Duisburg-Essen, 45147 Essen, Germany; 18 Institute for Experimental Immunology and Imaging, University Hospital Essen, University of Duisburg-Essen, 45147 Essen, Germany; 19 Department of Biospectroscopy, Leibniz Institut für Analytische Wissenschaften (ISAS) e.V., 44227 Dortmund, Germany; 20 Department of Anatomy, College of Medicine, Howard University, Washington, DC 20059, U.S.A.; 21 Department of Neurology, Nanfang Hospital, Southern Medical University, Guangzhou 510515, Guangdong, China; 22 Department of Biomedical, Surgical and Dental Sciences, Milan State University, 20122 Milan, Italy; 23 Department of Neurology, Eberhard Karls University Tübingen, 72074 Tübingen, Germany; 24 Brain Center, University Medical Center Utrecht, 3584 CX Utrecht, Netherlands; 25 Nesmos Department, Faculty of Medicine and Psychology, Sapienza University, 00198 Rome, Italy; 26 Department of Physiology, Istanbul Medeniyet University, 34700 Istanbul, Turkey; 27 Department of Surgery and Medical-Surgical Specialties, University of Catania, 95123 Catania, Italy; 28 Clinical Neurophysiology Research Unit, Oasi Research Institute IRCCS, 94018 Troina, Italy; 29 Institute of Stroke and Dementia Research, Ludwig Maximilians University Munich, 81377 Munich, Germany; 30 Department of Neurology, University Medical Center Hamburg-Eppendorf, 20246 Hamburg, Germany; 31 Department of Biomedical, Metabolic and Neural Sciences, University of Modena and Reggio Emilia, 41125 Modena, Italy; 32 Azienda Ospedaliero Universitaria di Modena, 41126 Modena, Italy; 33 Department of Neurology, St. Joseph Hospital Berlin-Weißensee, 13088 Berlin, Germany; 34 Department of Clinical Neurosciences and NIHR Biomedical Research Centre, University of Cambridge, CB2 0PY Cambridge, UK; 35 Department of Metabolism, Digestion and Reproduction, Imperial College London, W12 0NN London, UK; 36 Department of Pathophysiology, Medical University of Lublin, 20-090 Lublin, Poland; 37 Center for Clinical and Experimental Medicine, University of Medicine and Pharmacy Craiova, 200349 Craiova, Romania; 38 Department of Animal Biology, School of Biology, College of Science, University of Tehran, Tehran 14155-6465, Iran; 39 Biomedical Engineering & Biotechnology Department, Khalifa University of Science and Technology, Abu Dhabi 127788, United Arab Emirates; 40 Departments of Biological and Biomedical Sciences and Vision Science, Glasgow Caledonian University, G4 0BA Scotland, UK; 41 Department of Neurology, Institute of Clinical Medicine, University of Eastern Finland, 70210 Kuopio, Finland; 42 Center for Advanced Medical and Pharmaceutical Research, George Emil Palade University of Medicine, Pharmacy, Science, and Technology of Târgu Mureș, 540142 Târgu Mureș, Romania; 43 Department of Neurology, Brain Research Center, Sun Yat-Sen Memorial Hospital, Sun Yat-Sen University, Guangzhou 510120, Guangdong, China; 44 Second Department of Neurology, Attikon University Hospital, School of Medicine, National and Kapodistrian University of Athens, 12462 Athens, Greece; 45 Fondazione Paolo Procacci, 00193 Roma, Italy; 46 Department of Physiology, Faculty of Medicine, Dokuz Eylul University, 35220 Izmir, Turkiye; 47 Department of Clinical Genetics, Human Genetics and Genome Research Institute, National Research Centre, Cairo 12622, Egypt; 48 State Key Laboratory of Chemical Biology, Shanghai Institute of Organic Chemistry, Chinese Academy of Sciences, Shanghai 200032, China

**Keywords:** Clinical endpoint, translation bottleneck, experimental models, neurological therapy, pharmacological therapy, translation concept

## Abstract

Recent progress in translational neuroscience has significantly advanced our understanding of neurological diseases. Research progress closely went in line with innovations in research methods, which have expanded our insights considerably beyond previous limits. However, despite the development of disease-modifying treatments, therapeutic options in brain diseases still lag behind fundamental discoveries in basic neuroscience. This perspective examines the factors that hinder clinical progress in translational neuroscience and provides solutions on how to overcome them. Editorial board members of *Exploration of Neuroscience* were interrogated about the most prominent challenges they see in translational neuroscience and about possible ways to overcome these issues. Key challenges were seen at the interface between experimental research and clinical studies by several members, both from the basic and applied neuroscience fields, which include the selection of appropriate study readouts and endpoints. The establishment of refined study endpoints, combined with biomarkers capable of predicting treatment responses in human patients, will be crucial for the successful clinical implementation of new therapies. Further obstacles were found in the standardization of experimental models, interventions, and assessments both in animals and humans, as well as in the development of personalized treatment strategies. These challenges can be addressed through more clearly defined experimental procedures that closely match clinical conditions and precision-based approaches that ensure efficient therapeutic responses. As a great opportunity, treatment options targeting pathophysiological processes in multiple brain diseases and disease processes in different organ systems were noted. Significant barriers remain in the funding of investigator-driven clinical trials through public research programs, as well as the education of translational and clinician scientists dedicated to clinical translation. Enhanced communication between experimental neuroscientists and clinicians, with a shared understanding and common language, will be essential for the success of future research endeavors.

## Recent developments in the treatment of brain diseases

Treatment options have made significant advances in numerous brain diseases in recent years. As a result, many conditions that previously lacked causative, disease-modifying therapies are now amenable to such treatments. In ischemic stroke, for example, reperfusion therapies meanwhile allow enabling the recanalization of occluded arteries. Mechanical thrombectomy and the extension of the boundaries of intravenous thrombolysis have greatly improved patient management, with a major impact on brain damage, neurological outcome, and stroke survival [1, 2]. In multiple sclerosis, immunomodulatory treatments that dampen brain inflammatory responses were shown to reduce disease relapses and disease progression, with beneficial consequences for the accrual of disability [3–5]. In Alzheimer’s disease, a devastating neurodegenerative condition, immune therapies targeting β-amyloid (Aβ) have recently been found to slow down cognitive decline and enhance daily life activities, although still to a moderate extent [6, 7]. Despite the progress made, significant neurological deficits persist in the vast majority of stroke patients [8], while neurodegenerative diseases still continue to progress in the long run [9] and problems with disability progression in multiple sclerosis remain [10].

Given these persisting clinical problems, the editorial board members of *Exploration of Neuroscience* were interrogated by the editorial office about the most prominent challenges they see in translational neuroscience and about possible ways to overcome them. Responses were collected. Text passages provided by various editorial board members were integrated into this paper, which was again circulated among them. Editorial board members subsequently vividly provided text amendments and changes, which were integrated into the presented manuscript that was again circulated for approval. Considering the broad composition of the group bringing together both basic and clinical neuroscientists, as well as clinical neurologists and psychiatrists, this paper provides a unique perspective on how to orient research in the translational neuroscience field.

## Research methods and technologies as drivers of translational neuroscience advances

Clinical progress has been facilitated by significant advances in both basic and translational neuroscience, as the group members noted. The evolution of experimental research over the past decades has not followed a linear trajectory, but has followed quantum leaps paralleled by the enormous development of research methods, most notably in the neuroimaging and molecular biology fields [11]. The spatial resolution of biological processes was, for a long time, restricted by the resolution limits of light microscopy. These limits were overcome by superresolution microscopy (including expansion microscopy) [12] and cryo-electron microscopy [13], allowing spatial resolution at molecular and even up to atomic levels. The analysis of physiological processes was greatly facilitated by intravital microscopy (e.g., multiphoton microscopy [14, 15]) and in humans by magnetic resonance imaging (MRI) and positron emission tomography (PET) [16].

Breakthroughs in genetics have profoundly reshaped our understanding of several neurodegenerative and neuromuscular diseases, not only enabling earlier and more accurate diagnoses, but also paving the way for targeted therapeutic strategies. Landmark examples include antisense oligonucleotide therapies and gene replacement approaches, such as those successfully developed for spinal muscular atrophy (SMA) [17] and metachromatic leukodystrophy [18]. Gene cloning by CRISPR/Cas9, chemogenetic and optogenetic tools meanwhile allow manipulating defined neuronal cell types and pathways with high precision [19, 20]. More recently, advances in transcriptomics and proteomics facilitated our understanding of cellular signaling networks [21, 22]. Metabolomics help elucidating structure-function relationships [23], while information from different imaging modalities (e.g., multilabel fluorescence microscopy and spatial proteomics) can be co-registered by multiplex imaging [24]. The processing of research data is substantially enhanced by artificial intelligence (AI; e.g., deep learning) strategies, allowing the extraction of features from large data sets that hitherto escaped the attention of scientists [25]. AI will greatly simplify and improve the diagnosis of brain diseases in expert systems in the future. Its clinical success will greatly depend on human capacities to train AI algorithms and of AI experts and clinical doctors to interpret AI findings. The possibility to share entire multiomics datasets and to perform multiple analyses by different groups further boosted research in a collaborative manner. The use of advanced multiomics approaches (NULISAseq, OLINK, Somascan) for biomarker assessments advanced our knowledge, yet without being translated into clinics until now.

## Challenges in clinical translation

Considering the significant advances in experimental research, progress in clinical medicine still lags behind, the group members found. Have we truly grasped the nature of brain diseases in the animal models we use in experimental neuroscience, which in many aspects do not fully replicate human disease conditions? In the ischemic stroke and Alzheimer’s disease fields, recent progress in clinical patient management was preceded by a myriad of randomized controlled phase II and III study failures. In ischemic stroke, several studies aimed at establishing neuroprotective drugs [26, 27]. Aspects of treatment timing, drug delivery across the blood-brain barrier, which efficiently prevents the brain accumulation of drugs, and dose selection are likely responsible for a large number of study failures [28, 29]. In Alzheimer’s disease, the majority of studies focused on the prevention of Aβ aggregation or plaque formation [9, 30], which is thought to represent a central component of disease pathogenesis. Yet, it actually turns out that the etiology of Alzheimer’s disease is still not entirely understood. Experimental findings meanwhile challenged the Aβ cascade hypothesis of Alzheimer’s disease (see section “Modeling of brain diseases in a clinically relevant way” in Table 1). New pathophysiological concepts have recently been explored, some of which focus on the cerebral microvasculature [31–33], inflammation [34–36] and chronic infection [37–39] as disease mechanisms. Further technological advancements in human imaging—not only through the development of higher-resolution CT and MRI machines and novel techniques such as photon-counting, but also through the integration of automated and advanced image-analysis tools—represent a powerful means to deepen pathophysiological insights into complex brain diseases, such as Alzheimer’s disease. These innovations also enable more effective patient stratification, helping to distinguish responders from non-responders across a wide range of therapeutic approaches.

## Modeling of brain diseases in a clinically relevant way

A particularly important limitation of animal research is the genetic and environmental homogeneity of animal models, in particular mouse models, which contrasts with the diversity found in human populations and can limit the translational relevance of findings (section “Modeling of brain diseases in a clinically relevant way” in Table 1). Homogeneity refers to the use of genetically inbred mice of the same sex and age (mostly young male mice), exposed to highly standardized nutritional (i.e., standardized chow), hygiene (i.e., specific pathogen free, SPF) and social (i.e., small uniform cages with few play items) environments with highly controlled temperature and light cycles. This homogeneity is designed to reduce variability in experiments and increase statistical power. Furthermore, animal models often represent monogenic conditions (e.g., in Alzheimer’s disease or amyotrophic lateral sclerosis, ALS). However, neurological diseases in humans are mostly polygenic and multifactorial in origin. Single-gene models or chemically induced disease states rarely capture the full complexity. Humans have a large genetic diversity including racial/ethnic variety. Disease processes in humans manifest and progress differently across sexes, ages, and ethnic groups and are modified and conditioned by a large number of environmental factors, life habits, nutrition, behavioral influences, and by comorbid diseases, which predispose to neurological disease development. None of these factors is reflected in uniform animal models. Several neurological diseases primarily affect elderly human subjects that suffer from age-related risk factors or comorbid diseases. Experimental studies in young, otherwise healthy rodents, which are often exclusively male, poorly reflect these disease conditions. On the other hand, they are still better than transgenic models, where pathology is artificially and unnaturally forced. It is therefore not surprising that experimental disease models insufficiently mimic human disease conditions and may foster the focus on less relevant pathophysiological concepts. Greater attention is needed in animal research to age and sex differences, as well as the role of risk factors and comorbidities. In human research focusing on disease prevention, large-scale longitudinal cohort designs are widely used. Similar designs in animals would require the use of outbred instead of inbred animal strains. Studies in outbred strains are rarely performed in animals due to the larger data variability that would be associated with larger sample size and high resource use. To account for multiple influence factors, specifically of environmental influences, so-called exposomes have been proposed for facilitating our understanding of several neurological diseases [40, 41]. Exposomes represent the totality of environmental exposures that people experience throughout their lives, and how those exposures relate to their health. The gene-time-environment hypothesis posits that disease onset occurs through an interaction of genes with environmental exposures during ageing, while the multistep model suggests that several hits, at least some of which could be environmental, are required to trigger disease onset, even in the presence of highly penetrant mutations of genetically inherited diseases [40].

In experimental research, human-derived *in vitro* systems like brain organoids, induced pluripotent stem cell (iPSC)-derived neurons, patient-derived xenografts or humanized animals recently allowed for patient-specific disease modeling and move the research toward more complex models. Using outbred animal populations, diverse genetic reference panels such as like the Collaborative Cross in mice [42, 43], incorporation of both sexes and different age cohorts coupled with varied environments into experimental design can be decisive factors in translational research. Advanced imaging and biomarker development can provide non-invasive tools to validate disease mechanisms and longitudinally track disease progression in humans. Computational neuroscience and AI-based modeling can help integrate data across species to identify conserved mechanisms that are more likely to translate. The use of human disease models in a dish, on the other hand, poses ethical questions, which still require collective resolution by the scientific community. There is a lack of standardization of experimental models (ensuring reproducibility and data comparability) between different laboratories in translational neuroscience. This lack impedes the transfer of findings between labs. Standard procedures and joint training in workshops allow increasing the consistency of experimental model systems [44]. Experimental studies in animals should rigorously consider the nature of exploratory versus confirmative assessments in preclinical studies to rule out that treatment strategies are translated into clinics based on preliminary findings, findings obtained in model system with low external validity, or studies prone to type II statistical errors (i.e., mistakenly rejecting the null hypothesis). Studies should be performed in at least two species from, if possible, at least two laboratories, and where available at least two animal models should be used for mimicking complementary aspects of disease pathology [45]. The impact of relevant comorbidities should be considered in confirmative and translational studies [29, 46].

An important shortcoming of animal models is the inability to reliably mirror adverse drug reactions. One prominent example of an opportunistic central nervous system (CNS)-infection occurring under different immunotherapies in multiple sclerosis is progressive multifocal leukencephalopathy (PML) [47]. This rare, but severe side effect was not forecast in mechanistic studies in animal models, and likewise thus far there is a lack of a reliable animal model of PML. Similar examples exist in other disease areas.

## Selecting meaningful clinical readouts in animals

The demonstration of therapeutic efficacy critically depends on well-defined clinical scales and tests with proven reliability and validity (section “Selecting meaningful clinical readouts in animals” in Table 1). In experimental studies, observer-based scales and tests are generally used for evaluating the therapeutic activity of treatments. Of note, the improvement of clinical symptoms is not sufficient for demonstrating therapeutic efficacy in human patients. Instead, treatments must bring meaningful daily-life improvements that outweigh possible treatment side effects to be acceptable for clinical use. For this reason, drug authorities usually request patient-centered disability endpoints [48]. There are finely tuned scores and tests available in animals able to discriminate disease severities with high confidence. Yet, the nature of evidence of these readouts fundamentally differs from human endpoints. Since these tests are symptom-oriented, not disability-oriented, a fundamental disparity exists between preclinical and clinical measures, complicating translation efforts [48]. Several group members pointed out that there is a lack of standardization in behavioral testing procedures in translational neuroscience, results not rarely being based on small or biased samples with limited reliability or validity. This lack impedes the transfer of research findings between laboratories. Standardized procedures and joint training may again enhance consistencies [44].

## Selecting meaningful clinical endpoints in humans

Not only the selection of experimental readouts, but also that of clinical endpoints in humans poses unresolved challenges (section “Selecting meaningful clinical endpoints in humans” in Table 1), the group members noted. While some outcome measures—such as survival in ALS—are undoubtedly objective, they are poorly compatible with the practical constraints of clinical trials, which require shorter observation periods and cost containment. Conversely, commonly used clinical scales often fail to meet fundamental validity and reliability standards, as highlighted, for instance, by the ALS Functional Rating Scale-Revised (ALSFRS-R) [49] or the expanded disability status scale (EDSS) [50]. A weakness of some scores is that they do not appropriately measure disease stage (e.g., unified Parkinson’s disease rating scale, UPDRS, in Parkinson’s disease [51]) or when they try to approximate it, they are liable to bias by chance (e.g., EDSS) or memory errors (e.g., “sum-of-boxes” in clinical dementia rating scale, CDR). Yet they continue to be widely adopted in the recent past.

Pharmacological trials in humans often use grossly granulated disability scales as primary endpoint [48]. A frequently used global disability scale is the modified Rankin scale (mRS), which has low granularity (score ranges from 0–6). Although the mRS was established for revealing therapeutic improvements when treating large vessel occlusion stroke, its few categories do not allow for detecting smaller yet highly relevant stroke outcome changes [52]. It also neglects mental health aspects in stroke outcome, for instance, fatigue or cognitive impairment. Large patient numbers are required to demonstrate statistically significant differences in disease outcome based on mRS scores [48]. Statistical sample size assessments in clinical trials are often optimistic regarding expected effect sizes. To validate drug actions, the European Medicines Agency (EMA) has a preference for binary outcomes (i.e., good versus poor recovery) over ordinal outcomes (i.e., absolute scores) (https://www.ema.europa.eu/en/documents/scientific-guideline/points-consider-clinical-investigation-medicinal-products-treatment-acute-stroke_en.pdf). The Food and Drug Administration (FDA), on the other hand, encourages patient-reported assessments, which weigh benefits based on patient judgments (https://www.fda.gov/media/77832/download). Binary endpoints are highly arbitrary in their cutoff definition. They reduce biological information and increase the study’s sample size. Both strategies cannot easily be reconciled in single clinical trials, since choices must be taken regarding the primary endpoint used.

Meaningful clinical endpoints should match as closely as possible to preclinical findings, evaluate benefits that are functionally meaningful in daily life, measure abilities that are tightly linked to neurological impairments and sufficiently grade results for reflecting treatment success in the target population, which clearly argues against gross scales [48]. The group members identified a major need for refined endpoints in future pharmacological trials. Patient-reported outcomes are so far underdeveloped in many neurological and psychiatric fields. They should carefully consider social aspects and aspects of quality of life. The development of refined clinical endpoints will crucially depend on deepened communication between study investigators and drug agencies. The latter should bring up the courage to go new ways. In human studies, endpoints should include ratings by clinicians, patients, and caregivers, which is done in few disease areas at present. An exception are epilepsy trials, in which patient and caregiver diaries noting seizure type and frequency are traditionally used as clinical endpoints for FDA approval. In the epilepsy field, electronic devices and longterm video-EEGs are meanwhile considered more objective and reliable in drug evaluation [53]. In certain clinical pathologies, such as tinnitus, the determination of clinical endpoints is particularly challenging, since these pathologies are associated with purely subjective symptoms for which there are still no objectively measurable correlates. Even though more difficult to evaluate, study endpoints should also include open response formats so that they are not restricted to predefined assumptions.

## Characterizing structural and functional tissue responses

Classical histochemical and molecular biological tools have provided valuable insights into tissue responses, treatment efficacy, and underlying mechanisms, particularly through measures such as tissue volume in ischemic stroke and neurodegeneration studies. However, these approaches face limitations in spatial resolution, temporal sensitivity, and the ability to capture cellular heterogeneity (section “Characterizing structural and functional tissue responses” in Table 1).

The advent of advanced *in vivo* imaging modalities like multiphoton microscopy, MRI, and PET has begun to address some of these limitations by enabling time-resolved analysis of disease progression and therapy response. Yet, these techniques are constrained by tissue penetration depth and spatial resolution, restricting their insights to specific brain regions and cellular environments. Modern hybrid imaging approaches may help to overcome this situation [54].

Recent breakthroughs in multi-omics approaches—including transcriptomics and proteomics—at the single-cell level have transformed our capacity for deep tissue phenotyping. These methodologies unveil cellular heterogeneity previously obscured in bulk analyses, exposing distinct cellular states, functional modules, and subtle differences in cellular behaviour that are critical for understanding complex tissue responses [21, 22]. Applying single-cell analyses in tissues allows us to identify and characterize previously neglected heterogeneities, such as distinct immune cell subsets, degenerated neurons, or regenerative cellular niches [21, 31]. These insights are crucial for making precise correlations between cellular states—such as activation, quiescence, or apoptosis—and tissue features like inflammation, degeneration, and regeneration. This granular understanding enhances the ability to link cellular phenotypes directly to functional tissue outcomes and disease processes. The integration of single-cell transcriptomics and proteomics within disease models opens new avenues for extracting novel information on disease mechanisms and therapeutic targets. It facilitates the identification of cellular functional modules associated with specific pathological features, enabling the development of more precise, mechanism-based interventions. Single cell multiomics allow dissecting cell biological processes with unprecedented precision and information depth, and have the potential to become a standard research language in neuroscience [21]. At the same time, this will require the development of conceptual frameworks and a solid understanding of how to integrate data in a meaningful way for clinical applications, as the group members also stated.

Of note, neurological diseases in many cases are not purely CNS disorders, but have underlying systemic, multi-organ mechanisms that contribute to disease development. Examples include the role of immune responses in ischemic stroke, neuroinflammatory and neurodegenerative diseases, which turned out to be promising targets for immunomodulatory treatments in the recent past [55]. Remote organ interactions (e.g., brain-heart axis, brain-gut axis, brain-liver axis) were found to modulate disease recovery processes as well as the brain’s responses to drugs [56, 57]. Immune mechanisms, extracellular vesicle-mediated processes, and sympathetic/parasympathetic nerve systems are key drivers of remote organ interactions [56]. In the gastrointestinal system, the microbiome was found to have a crucial impact on immune responses, which critically influences enteric nervous system (ENS) function [58] and CNS disease [59]. There is a lack of standardized methods to reliably assess remote organ interactions (e.g., with the heart or gastrointestinal system) in brain disease models. Future studies will have to define toolboxes enabling a proper classification of organ networks providing a framework for meaningful translation studies.

As a shortcoming with particularly serious consequences for translation success, there is a lack of biological readouts enabling the assessment of possible adverse drug actions in translational neuroscience. Some side effects (e.g., brain hemorrhages after ischemic stroke) are difficult to assess in small animal models or require dedicated focus to detect them [60]. Also, subtle evidence needs to be taken seriously, since it may have significant implications for patients [60]. In many cases, side effects that initially seemed minor later resulted in devastating disease outcomes of clinical trials [61], outcomes that could have been anticipated based on prior animal studies [62]. Possible interactions with drugs, including drugs administered for other medical conditions, need to be considered carefully. An example is laxatives, which have multiple interactions with other drugs [63]. The scientific community will have to develop more refined concepts to detect safety risks and side effects of new treatments. For ensuring the rigidity of data, publication media should ensure that research adheres to established guidelines (e.g., STAIR, STEPS, RIGOR) [45, 64] via upload of checklists along with publications.

## Bridging experimental and clinical studies by biomarkers

In the translation of research findings from the bench to the bedside, biomarkers are a powerful tool to evaluate the successful transfer of a therapeutic concept from one species to another, and from animals to patients, informing about the biological effects of an intervention. A major advantage of biomarker-centered phase IIa clinical studies is that they typically require much smaller patient cohorts than phase IIb and phase III efficacy trials, which are also significantly more expensive, longer in duration and complex. Phase IIa studies are particularly suitable for linking experimental and clinical studies. A good example is phase IIa trials in multiple sclerosis based on MRI outcome parameters. Another example is photoparoxysmal EEG responses as outcome in phase IIa epilepsy trials [65]. Successful phase IIa biomarker studies, however, do not exclude that treatments successful in biomarker studies subsequently fail in phase III clinical endpoint trials [66]. In translational neuroscience, the lack of biomarkers that bridge preclinical and clinical studies is a major bottleneck that hampers research progress (section “Bridging experimental and clinical studies by biomarkers” in Table 1), as group members noted. In many neurological conditions, we lack direct access to the brain, making it difficult to validate pathomechanistic hypotheses derived from animal models in small-sized human studies. As a result, large, expensive phase IIb and phase III studies are then required to assess clinical efficacy in the absence of intermediary biomarkers. Surrogate markers in the cerebrospinal fluid (CSF) or blood may help overcoming existing limitations related to the unavailability of brain tissue sampling. Among these, immune and protein-based biomarkers in the CSF and blood show great promise in bridging the gap between experimental and clinical studies and testing the validity of therapeutic concepts [67, 68]. Besides clinically established markers such as CSF Aβ and tau protein [6, 67, 69], neurofilaments, specifically neurofilament light chain (NfL) levels, are emerging as robust biomarkers of neurodegeneration with a growing role in both patient stratification, disease progression, and therapeutic response monitoring [70–72]. miRNAs have also been studied, mostly in experimental and recently also in disease contexts [73]. Their clinical value still remains to be shown. In addition to CSF and blood biomarkers, MRI (e.g., chemical exchange saturation transfer MRI, CEST-MRI [74, 75]) and PET (e.g., Aβ or tau PET [76]) techniques allow providing non-invasive information about brain disease processes, including brain metabolism (e.g., CEST-MRI) or molecular pathologies (e.g., Aβ or tau PET), which can replace direct brain tissue measurements and can be used as patient stratification biomarkers. While Aβ and tau PET are already clinically established in Alzheimer’s disease management [76], the predictive value of several other imaging biomarkers remains to be scrutinized.

A huge obstacle in brain diseases that impedes the successful implementation of treatment concepts is the exceedingly long time required to move from a promising research finding to a clinical therapeutic or drug. Considering advances of patient management, this long time span carries the risk of obsolescence in fast-evolving research fields. In case of rare diseases, the variability of disease outcomes and low reproducibility of treatment actions impose clinical translation challenges. Patient post-approval registries are the procedure of choice to overcome shortcomings of statistical power in such clinical settings.

## Challenges associated with research findings from single laboratories or single clinical departments

A serious pitfall in translational research is study bias attributed to research findings in single laboratories or clinical environments, which cannot be replicated in other places (section “Challenges associated with research findings from single laboratories or single clinical departments” in Table 1), the group members furthermore found. Promoting collaborative consortia in experimental research (e.g., bicenter or multicenter preclinical studies, studies involving more than one species and animal model) and clinical research (e.g., NIH BRAIN Initiative, Human Connectome Project, ENIGMA Consortium) helps to validate treatments across different models and populations. The availability of large datasets is another driver for neuroscience evidence validation and replication (e.g., UK Biobank, ADNI, ABIDE). Precision medicine approaches using genomics, transcriptomics, and neuroimaging to stratify patients into biologically relevant subgroups will be the way of the future. Big data and machine learning will be a tool to identify hidden patterns across patient data that can inform new diagnostic or therapeutic targets. However, such data analysis should not neglect the nature and quality of the underlying data source. This can be an issue in neuroepidemiology, where large sets of poorly characterized data carry the risk that data are not up to the task [77]. Data quality issues can significantly distort scientific outcomes in neuroepidemiology, as has been demonstrated [78, 79], which may yield wrong leads for clinical testing. A novel way on how to conduct clinical trials is the development of adaptive clinical trial designs that allow treatments to be tailored or adjusted based on individual patient responses. This strategy allows the involvement of highly heterogeneous brain diseases or patient populations that have seen insufficient involvement in clinical research in the past. Multicenter or community-based research can ensure that underrepresented and diverse populations are included, improving research generalizability.

## Scientific networks as clue to the successful implementation of new research concepts

Unfortunately, due to recent political developments, research funding in some countries (e.g., U.S.A.) has seen significant cuts or, in some cases, has been completely cancelled, which a number of group members identified to represent a major challenge in their field (section “Challenges associated with research findings from single laboratories or single clinical departments” in Table 1). The NIH BRAIN Initiative, for example, had already experienced substantial reductions of funding in the recent past and now again faces significant cuts. The Human Brain Cell Atlas of the NIH BRAIN Initiative is designed to understand the complexity and heterogeneity of human brain cell types to improve disease modeling and therapeutic targeting. The NIH BRAIN Initiative has funded a comprehensive project to map the diversity of human brain cells, resulting in a detailed atlas that identifies many cell types across various brain regions. This atlas enhances our understanding of the cellular composition of the human brain and its relation to neurological disorders. Since the atlas serves as a critical tool for researchers aiming to develop precise interventions for brain diseases, which facilitate the translation of basic research into clinical applications, the atlas is another means to address and overcome challenges in translational neuroscience. It would be a major damage to the scientific community if such activities could not be continued in the longer run.

Collaborative projects require larger scale research funding. The German Research Council (Deutsche Forschungsgemeinschaft, DFG), for example, supports Collaborative Research Centers (CRC) or Research Groups involving researchers across the country that support both basic and translational neuroscience projects over time-windows of up to 12 and 8 years, respectively. This type of funding enables larger scale research endeavors in defined research fields. An example is the CRC TRR332 “Neutrophils: Origin, fate and function” (www.neutrophils.de), which comprises research activities in the polymorphonuclear neutrophil field that bridge basic and translational research including neuroscience using advanced multiomics tools combined with clinically relevant disease models and functional assays. By generating research synergies, collaborative research projects of different universities can create particular dynamic research environments. One example to address problems in translational neuroscience is the NeuroTech Harbor Howard – Johns Hopkins partnership. NeuroTech Harbor (https://neurotechharbor.org/) is a collaborative technology accelerator between Howard University (Washington, D.C.) and Johns Hopkins University (Baltimore, MD) funded by NIH through the Blueprint MedTech program. It aims to expedite the development of medical devices for diagnosing and treating neurological disorders such as Alzheimer’s disease, Parkinson’s disease, stroke, and addiction. The initiative emphasizes inclusivity by supporting underrepresented innovators and ensuring that developed technologies are accessible to diverse communities. It would matter significantly, if such programs had to be stopped due to discontinuation of research funding.

## Development of personalized treatment concepts

Customized personalized treatments employing precision medicine approaches are the strategy of choice in heterogeneous therapeutic areas, in which “one-size-fits-all” approaches are not feasible or unlikely to be effective. A typical example of a therapy which is widely administered in such areas is non-invasive brain stimulation (NIBS) [80–82]. The heterogeneity of diseases poses therapeutic challenges (section “Development of personalized treatment concepts” in Table 1). Herein, advanced neuroimaging (e.g., fMRI, voxel-based lesion analyses) and neurophysiological assessments (e.g., high-density EEG) can be used to identify specific neural circuit dysfunctions in individual patients as a basis for personalized therapy [83, 84]. Computational modeling can help defining stimulation parameters (e.g., target location, intensity, frequency, duration) to optimally modulate these circuits. In this complex but fascinating scenario, any biomarker able to predict treatment responses will also help in selecting the most appropriate NIBS protocol for each patient. Of note, the targeted stimulation of certain, narrowly defined brain structures is often possible only to a limited extent. Even if there are strong hypotheses for a potential mode of action, technical barriers not rarely impede the clinical implementation of NIBS strategies.

While invasive deep brain stimulation is meanwhile well established in conditions like Parkinson’s disease [85, 86], establishing reliable and long-lasting therapeutic effects is often challenging in NIBS, and immediate effects of stimulation may not always translate into sustained clinical improvements (section “Development of personalized treatment concepts” in Table 1). Factors such as the chronicity or duration of the disorder, age- and sex-related differences, the occurrence of compensatory mechanisms within the brain, the interaction with other treatments (both pharmacological and non-pharmacological), as well as the timing of stimulation can influence the impact and duration of NIBS effects [87, 88]. A solution to these challenges might be the combination of NIBS with other therapeutic modalities, such as pharmacological therapy, psychotherapy, cognitive training, virtual reality [89] or augmented reality [90], which enhance and prolong its effects or which shift disease processes from disease chronification to resolution. Importantly, in the case of NIBS, treatment protocols may have to be optimized for frequency, intensity, duration, and number of sessions depending on target sites, based on a deeper understanding of neuroplasticity mechanisms. Exploring any maintenance stimulation strategy or designing “booster” sessions is also essential to ensure more sustained treatment benefits [91].

Clinically, despite growing evidence for the efficacy of NIBS in some neurological (e.g., stroke) and psychiatric conditions (e.g., major depression), the widespread translation of research findings into daily clinical practice is still limited [92]. Challenges include the heterogeneity of clinical conditions, the lack of standardized treatment protocols, and the need for specialized equipment and trained personnel [93]. Once established in clinical trials, evidence-based clinical guidelines and protocols are needed in specific diseases, as well as rigorous cost-effectiveness analyses demonstrating the long-term benefits of therapeutic strategies (thus also providing support for reimbursement by healthcare providers). Training programs for clinicians are also required to promote the user-friendly access of clinicians to such treatments.

## Disease-overarching biological principles or therapeutic activities as opportunity for treatment development

As an important overarching theme in brain diseases, the group members identified shared disease mechanisms that are common to a wide range of disease groups (section “Disease-overarching biological principles or therapeutic activities as opportunity for treatment development” in Table 1). An aspect, for example, which bridges neurodegenerative diseases and major depressive disorder, is subtle neuroinflammation [94, 95], while in ischemic stroke and traumatic brain injury, proinflammatory responses are a major driver of evolving brain injury [55, 96]. Moreover, inflammation-associated remodeling of the brain’s extracellular matrix represents a crucial, yet underexplored component of the pathophysiology of ischemic stroke, Huntington’s disease, and Alzheimer’s disease [97]. Proinflammatory responses exacerbate injury severity in a wide range of cells and tissues. Thus, the immune system gets locked in a prolonged defensive state, unable to resolve chronic inflammation [98]. Remarkably, strikingly similar proinflammatory responses have been noted in hereditary brain diseases and acquired neurodegenerative diseases. In all settings, neuroinflammatory processes drive disease progression and outcome. One aim of cell-based therapies is to resolve proinflammatory responses, helping to shift the immune system into a regulatory state [96]. In several diseases, such as stroke, Alzheimer’s disease and multiple sclerosis, this shift is essential for enabling neuroplasticity responses and brain repair [55, 98]. Intriguingly, the underlying cell biological principles that control immunity are conserved across species: Basic principles can already be found in *Drosophila melanogaster* [99, 100]. At first glance, these cross-disease similarities may seem surprising. However, with a deeper look into developmental biology, it gets apparent that biological systems reuse core signaling pathways and molecular decision-makers to prime tissues for recovery [56].

Shared mechanisms may not only underlie different brain diseases, but also extend to other organs that exhibit diseases with very similar disease mechanisms. Of note, highly prevalent diseases, such as ischemic stroke, myocardial infarction, heart failure, and chronic kidney disease (CKD), are complex disorders, which have multiple etiologies that are shared by these different disease entities [56]. Several disease processes are linked to underlying macroangiopathy or microangiopathy. There are shared cell signaling pathways jointly activated in these disease conditions. By targeting these signaling pathways, several pharmacological treatments have recently been identified (e.g., SGLT2 inhibitors, such as dapagliflozin and empagliflozin; GLP-1 agonists, such as semaglutide), which have impressive activity in several disease states, enhancing long-term disease outcomes [56]. Besides the cardiovascular system, the gastrointestinal system, including the ENS, reveals a large number of organ interactions that profoundly modify CNS disease processes [101, 102]. Disease overarching pathophysiological principles have great potential for the development of new therapies, as the experience gathered in one disease area can be translated to another one. The successful implementation of new therapies requires the communication of clinical neuroscientists with colleagues in other medical areas, such as cardiology or nephrology. Not rarely, this mutual communication opens the way for the repurposing of drugs, which can now be evaluated in additional disease contexts.

## Getting well-designed proof-of-concept studies going

Having said this the authors agreed that it is now up to applied neuroscientists, clinician scientists, neurologists, and psychiatrists to initiate well-designed proof-of-concept studies that bridge the gap in the translational neuroscience field. The need of multicenter studies necessitates robust research financing, without which the successful translation of research concepts becomes unattainable. There was consensus in the group that the need of cost-saving in translational neuroscience should not be realized at the expense of collaborative multicenter studies, which are able to provide strong evidence for ultimately promising treatment targets. Disease-overarching biological principles or therapeutic activities particularly strongly argue in favor of the clinical implementation of a given treatment concept, since observations made in one clinical condition may instruct concepts in related ones. There is a lack of adequate funding for larger scale animal preclinical randomized controlled trials (RCTs) in translational neuroscience, and motivating young investigators to conduct these trials, which offer limited scientific merits, turns out challenging. Due to the inability to recruit patients during the COVID-19 pandemic, several clinical RCTs were unable to be completed, leading to current hesitancy in the venture capital sector to fund clinical proof-of-concept trials, which is currently a huge challenge. Translational neuroscience is still considered very challenging by the pharmaceutical and biotech industries, which are still mindful of the previous failed studies, e.g., in stroke and Alzheimer’s disease. Part of this pessimism stems from the challenges imposed by the blood-brain barrier that impedes the brain entry of blood-borne factors, including intravenously administered drugs. Luckily, with recent success in the implementation of new therapies, e.g., in stroke [1, 2] and Alzheimer’s disease [6, 7], these previous failures have been overcome at least to some extent.

Additional relevant aspects relate to scientific integrity and research publishing. Central data bases should make available data sets from experimental and clinical studies to other scientists for subsequent analyses. Platforms like Open Science Framework should be used more systematically to facilitate translational collaborations in designing, analyzing, evaluating, and publishing research. Open-access publications should include raw data, so that data can be merged more easily into meta-analyses or can be efficiently reused for other research questions. This research strategy is highly cost-effective. Scientific integrity has recently been challenged by predatory journals. With the implementation of open access publishing in scientific journals, there have been tremendous increases of publication costs in many scientific journals in recent years, which by far exceed the actual costs of publishing. Research bodies should provide support for open access publishing with stringent peer review and quality criteria, but at the same time insist that publishers do not impose out-of-scale prices, which undermine the possibilities of lower income countries to get their research published. It is the firm belief of the authors that worldwide interaction and collaboration, which includes developed and developing countries, will be a major driver that fosters translational research success to the benefit of all patients around the globe.

## Figures and Tables

**Table 1.** Prominent challenges in translational neuroscience and strategic solutions

Translation aspect	Challenges	Solutions
Modeling of brain diseases in a clinically relevant way	Prevailing disease concepts (e.g., Aβ cascade hypothesis of Alzheimer’s disease [6, 7]) impede focus of additional disease pathomechanisms, which remain understudied but might represent more promising therapeutic targets Uniform and monogenic disease models do not reflect multifactorial and polygenetic nature of human diseases (e.g., transient intraluminal middle cerebral artery occlusion for ischemic stroke, transgenic Alzheimer’s models) Homogeneity of laboratory animals, which are typically inbred, young, male and otherwise healthy. Homogeneity contrasts human genetic diversity, age and risk factors, and comorbidities of human patients Animal genetics does not predispose to human disease processes Animal models do not mimic life habits, nutritional, hygiene and social environments of humans Lack of standardization of animal models between research laboratories	Elucidate additional pathomechanisms and their utility as treatment targets (e.g., role of microvasculature [31–33] and inflammation [34–36] in Alzheimer’s disease) Use more complex disease models (e.g., thromboembolic model of ischemic stroke, Alzheimer’s models that involve vascular pathology) Use of outbred animals, aged animals, animals of both sexes, animals with risk factors and comorbidities [45, 64], animals from diverse genetic reference panels (e.g., Collaborative Cross) [42, 43] Use of human organoids, iPSC-derived neurons, patient-derived grafts or humanized animals as research objects Model environmental factors in animals (e.g., enriched environments), consider so-called exposomes [40, 41] in data analysis Standardized procedures, joint training in workshops [44]
Selecting meaningful clinical readouts in animals	Observer-based symptom-oriented clinical (neurological/psychiatric) scales/tests in animals do not mimic patient-centered disability endpoints in humans; daily-life relevance mostly unclear Animal studies frequently based on small or biased cohorts that are insufficiently powered Lack of standardization of animal behavioral testing procedures between laboratories	Disparity cannot easily be resolved [48], tests in animals should as closely as possible evaluate daily-life relevant contents Adequately powered cohorts, stringent randomization and blinding Standardized procedures, joint training in workshops
Selecting meaningful clinical endpoints in humans	Clinical scores do not appropriately measure disease stage (e.g., UPDRS) or are liable to bias by chance (e.g., EDSS) or memory errors (e.g., CDR), which limits data reliability/validity Use of grossly granulated scales unable to reveal fine improvements (e.g., mRS) Randomized controlled trials often postulating optimistic effect sizes of treatments	Refine scales or develop patient-centered tools, for which reliability/validity is thoroughly tested Develop more finely granulated scales in interaction with drug authorities (i.e., FDA, EMA) More realistic effect size assessments, which require larger studies
Characterizing structural and functional tissue responses	Classical histochemical and molecular biological tools face limitations in spatial resolution and temporal sensitivity Classical histochemical and molecular biological tools unable to capture cellular heterogeneity Brain tissue assessments neglect systemic disease processes Brain tissue assessments frequently neglect undesirable side effects	*In vivo* imaging (e.g., multiphoton microscopy [14, 15], PET [16]) enables time-resolved assessments, superresolution microscopy [12] and cryo-electron microscopy [13] exceeds spatial resolution limits Single cell multiomics allows deep tissue phenotyping, linking cell phenotypes and functional states [21, 22]; need of conceptual data integration framework Characterize systemic immune involvement and remote organ interactions (e.g., brain-heart axis, brain-gut axis) Refined concepts to detect safety risks and side effects
Bridging experimental and clinical studies by biomarkers	Frequent lack of brain biomarkers in humans capable to validate pathophysiological concepts in phase IIa studies before large scale efficacy trials	Reinforce biomarker development, search for non-invasive biomarkers, replace brain biomarkers by CSF or blood biomarkers where adequate, select promising treatments for clinical translation based on suitable biomarker existence
Challenges associated with research findings from single laboratories or single clinical departments	Single center research findings carry risk of lack of replication in other places due to lab-specific model or center-specific patient characteristics Single center studies restricted regarding animal or patient numbers recruited, data sets of moderate size providing gross efficacy assessments only Highly heterogeneous brain diseases or patient populations underrepresented in clinical trials, particularly in single center studies Funding of multicenter studies recently saw significant cuts in some countries due to political developments, which put at risk collaborative research activities	Collaborative multicenter consortia able to validate concepts across models or populations Multicenter studies allow identifying hidden patterns in large data sets, enabling refined efficacy assessments Multicenter, including community-based, studies can ensure that diverse populations are included, improving research generalizability. Adaptive trial designs allow treatment tailoring Continuation of multicenter and collaborative research funding
Development of personalized treatment concepts	Heterogeneity of diseases, which precludes “one-size-fits-all” approaches, poses therapeutic challenges Immediate effects observed in single patients not always translate into sustained clinical improvements Widespread translation of treatments into clinical practice often limited [92], need for specialized equipment and trained personnel posing challenges [93]	Advanced neuroimaging (e.g., fMRI) and neurophysiological assessments (e.g., high-density EEG) can help identifying therapeutic targets amenable for personalized therapy [83, 84] Chronicity of disorder, age and sex differences, compensatory mechanisms, interaction with other treatments, and treatment timing need to be taken into account; combination with other therapeutic modalities (e.g., pharmacotherapy, psychotherapy, cognitive training) may allow sustained responses Evidence-based guidelines and standardized protocols, rigorous cost-effectiveness analyses, user-friendly training programs
Disease-overarching biological principles or therapeutic activities as opportunity for treatment development	Highly subdivided therapeutic landscape impedes larger scale progress in translational neuroscience Targeting the brain insufficient in diseases with strong systemic pathophysiology or diseases exhibiting strong remote organ (e.g., brain-heart) interactions	Choose shared disease mechanisms that are common to a wide range of brain diseases as therapeutic target (e.g., proinflammatory responses associated with neurodegenerative processes) Treat systemic disease process that underlies the brain pathology (e.g., cardiac dysfunction, macroangiopathy or microangiopathy in stroke)

## Abbreviations

AI: artificial intelligence
ALS: amyotrophic lateral sclerosis
Aβ: β-amyloid
CNS: central nervous system
CRC: Collaborative Research Centers
CSF: cerebrospinal fluid
EDSS: expanded disability status scale
ENS: enteric nervous system
FDA: Food and Drug Administration
iPSC: induced pluripotent stem cell
MRI: magnetic resonance imaging
mRS: modified Rankin scale
NIBS: non-invasive brain stimulation
PET: positron emission tomography
PML: progressive multifocal leukencephalopathy
RCTs: randomized controlled trials
